# Establishment and Optimization of a Dynamic Model for Energy and Protein Requirements in Meat Ducks

**DOI:** 10.1016/j.psj.2026.106443

**Published:** 2026-01-12

**Authors:** Zhengbo Li, Qiong Liu, Dingtao Peng

**Affiliations:** a School of Mathematics and Statistics, Guizhou University, Guiyang 550025, Guizhou, China; b School of General Education, Moutai Institute, Renhuai, Guizhou 564500, China

**Keywords:** Meat ducks, Energy requirements, Protein requirements, Dynamic model, Nutrient utilization

## Abstract

The efficient utilization of energy and protein resources is crucial for the growth and development of meat ducks, as well as for the profitability of the poultry industry. This study aims to establish and optimize a dynamic model for the energy and protein requirements of meat ducks. A series of experiments were conducted with different dietary energy and protein levels. The growth performance, carcass quality, and nutrient utilization of the meat ducks were comprehensively evaluated. The results showed that when the dietary metabolizable energy (ME) was 12.8 MJ/kg and the crude protein (CP) level was 17.5%, the meat ducks exhibited the best growth performance and feed conversion ratio. The established dynamic model takes into account the interactive effects of energy and protein on the growth and metabolism of meat ducks, incorporating factors such as environmental temperature and breed characteristics. It provides a scientific basis for formulating precise feeding strategies to meet the nutritional needs of meat ducks at different growth stages, thereby improving production efficiency and reducing feed costs in the meat duck industry. The optimization of the model also considers the practical application in different production environments and duck breeds, enhancing its adaptability and reliability.

## Introduction

Precise nutrition supply in meat duck breeding is crucial for improving production efficiency and resource utilization (Yanhua et al., 2025). However, existing energy and protein requirement models for meat ducks are often based on static growth stages or single environmental conditions, failing to meet the demands of dynamic production practices (Yanyun et al., 2024). For instance, the protein requirement for Cherry Valley ducks can be 35% higher in winter than in summer, yet traditional models overlook such variables and inadequately quantify energy-protein interactions (Shaomeng et al., 2025).

The global meat duck industry faces dual challenges of resource efficiency and economic benefits. China accounts for over 75% of the world's meat duck production, yet nutritional standards for imported breeds often misalign with local production modes (Weijuan et al., 2022). Although China's three-stage feeding method reduces management complexity, it lacks systematic quantification of dynamic energy and protein demands at each stage (Huimin et al., 2024). A trial involving 8,540 duck houses revealed that imported standard formulas led to 12–15% higher body weights in winter compared to locally optimized schemes, while summer heat stress caused an 18% protein waste rate (Lijun et al., 2024). The limitations of static models exacerbate resource waste and suppress the genetic potential of meat ducks.

Existing factorial models attempt to address dynamic demands but have inherent flaws. For example, while seasonal adaptations partially account for metabolic maintenance requirements (e.g., 0.305 kJ/kg W⁰·⁷⁵ in winter), they fail to integrate energy-protein synergies, such as the 23% improvement in feed efficiency at energy-protein ratios of 125–135:1 (Wenxuan et al., 2024). Moreover, these models often overlook the dynamic metabolic characteristics of growth stages and breed-specific needs. The protein deposition rate in juvenile ducks (0–21 days) is 40% higher than in later stages, making them more sensitive to energy concentration (Huang, 2024). At the same energy level, protein deposition efficiency in Muscovy ducks is 12–15% lower than in Cherry Valley ducks, yet most model parameters derive from single-breed trials, leading to a 20% error rate in cross-breed applications (Jiayin, 2024).

Therefore, constructing a dynamic energy-protein synergy model that integrates environmental dynamics, growth stages, and breed-specificity is critical. This study introduces bivariate coupled equations incorporating metabolic weight and live weight gain, utilizing multi-seasonal and phased mass production data. By optimizing nonlinear parameter fitting with machine learning algorithms, this research aims to fill the gap in dynamic nutrition modeling, provide theoretical support for precise feeding systems, and promote resource-efficient animal husbandry.

## Literature Review: Research Progress and Challenges

### Core Parameters and Controversies of Energy Demand

Accurate determination of energy requirements is vital for optimizing breeding efficiency and ensuring animal health. Key parameters include maintenance energy requirements (MER) and growth energy requirements (GER). MER, the energy needed for basic life functions, varies with growth stages (Wenpeng, 2024). For instance, young meat ducks have higher MER per unit weight due to rapid growth and metabolism (Shanglin et al., 2024). GER, used for tissue growth and fat deposition, also varies by breed; fast-growing breeds require more efficient energy conversion (Juan et al., 2024).

Existing factorial models attempt to address dynamic demands but have inherent flaws. For example, they fail to integrate energy-protein synergies and often overlook the dynamic metabolic characteristics of growth stages and breed-specific needs. (Yu, 2023). However, consensus on the optimal ratio for different growth stages is lacking. While ruminant studies offer insights into energy metabolism and protein utilization (Yan, 2023), translating these findings to meat ducks requires further exploration.

Advanced techniques like stable isotope tracing and gene expression analysis offer precise insights into energy metabolism but are limited by high costs and operational complexity (Zhifeng, 2023). Traditional feeding trials, though intuitive, suffer from environmental and feed quality variabilities (Fanti, 2023).

### Dynamic Characteristics of Protein Requirements

Protein requirements in meat ducks are influenced by environmental factors, breed characteristics, and physiological states. Seasonal variations affect feed intake and protein distribution; summer heat stress reduces intake, necessitating higher dietary protein concentrations, while winter cold increases energy demands for thermoregulation, altering protein utilization (Ke, 2023).

Breed differences are significant; meat ducks and layer ducks have distinct protein requirement models due to differing production purposes and physiological traits (Bingxue, 2023). Faster-growing meat duck breeds exhibit higher protein demands.

Existing protein models, often based on factorial approaches (e.g., CPR = a·BGW + b·W⁰·⁷⁵), typically ignore seasonal fluctuations and amino acid synergies, limiting their predictive accuracy under practical conditions (Yuning, 2023). Ruminant research provides valuable methodologies for dynamic nutrient modeling, emphasizing the need to consider growth stages and environmental factors (Baoxing, 2023).

Physiological states (e.g., reproduction) further complicate protein requirements, yet systematic studies on meat ducks under varying physiological conditions are scarce (Yinan, 2023). Advanced methods, such as indicator amino acid oxidation, could enhance the accuracy of protein requirement determinations (Xuesheng et al., 2023).

In summary, research on meat duck energy and protein requirements faces challenges in core parameter determination, nutrient interactions, and methodological limitations. Constructing robust models necessitates integrating factors like growth stage, environment, breed, and physiological state.

### Summary of Existing Mathematical Models and Their Limitations

To quantitatively predict the energy and protein requirements of meat ducks, several mathematical models have been developed in previous studies. These models can be broadly categorized, and their characteristics are summarized in Table 1 below.

**Table 1:** Comparison of existing mathematical models for nutrient requirements in meat ducks.

Model Type	Representative Equation (s)	Key Advantages	Inherent Limitations / Disadvantages	References
Static / Phase Feeding Models	ME (or CP) = Constant value (for a specific growth phase, e.g., starter, grower, finisher)	- Simple to implement and manage. - Low computational requirement.	- Fails to account for continuous physiological changes within a phase. - Ignores environmental and individual variability. - Leads to under- or over-feeding.	(Huimin et al., 2024), (Yuning, 2023)
Factorial Models	Requirement = Maintenance + Production e.g., ME = a·W^0.75 + b·BGW CP = c·W^0.75 + d·BGW	- Mechanistically sound framework. - More physiological than static models.	- Parameters (a, b, c, d) are often static and derived from limited trials. - Lacks dynamic interactions (e.g., Energy-Protein synergy). - Oversimplifies environmental and breed effects (e.g., uses fixed seasonal factors).	(Wenxuan et al., 2024), (Yuning, 2023), (Baoxing, 2023)
Regression Models (Empirical)	Y = β₀ + β₁X₁ + β₂X₂ + ... (Where Y is performance, Xs are nutrient levels)	- Derived from experimental data, good fit within specific conditions. - Can identify key influencing factors.	- Extrapolation poor outside the range of original data. -Correlation does not imply causation. - Does not explicitly separate maintenance and growth needs.	(Fanti, 2023)
NRC (1994) and Other Standard Models	Provides tabulated values for requirements at different body weights.	- Authoritative reference, widely accepted. - Comprehensive review of available data at the time.	- Values are often averages and not dynamically adjustable. - Limited consideration for modern breeds, feeding systems, and environmental challenges. - Does not account for real-time inputs.	(Weijuan et al., 2022)

Note: This table synthesizes the primary model types identified in the literature. ME: Metabolizable Energy; CP: Crude Protein; W: Body Weight; BGW: Body Weight Gain.

The limitations outlined in Table 16 highlight the critical research gap. While factorial models provide a logical structure, their static parameters cannot adapt to the dynamic nature of duck metabolism influenced by real-time environmental temperature (ET), varying growth rates (BGW), and genetic potential (BC). Furthermore, the non-linear interaction between energy and protein, which is crucial for optimal nutrient utilization (Wenxuan et al., 2024), is not captured. The model established in this study directly addresses these shortcomings. It enhances the traditional factorial approach by introducing a multi-dimensional parameter system (W^0.75, BGW, ET, BC) and machine learning-optimized, dynamic coefficients, transforming it from a static calculation into a responsive, precision nutrition tool.

## Construction Method of The Dynamic Factorial Model

### Multi-dimensional Parameter System Design

The dynamic nutrient demand model transcends traditional single-variable frameworks by incorporating a four-dimensional parameter system: metabolic body weight (W⁰·⁷⁵), daily gain (BGW), ambient temperature (ET), and breed coefficient (BC). The energy requirement is modeled using a coupled equation:

(1) $$ME=\alpha \cdot dotW^{\left\{0.75\right\}}+\beta \cdot dotBGW+k_{3}\cdot dotET+BC\;\;($$

Metabolic body weight (W⁰·⁷⁵) serves as the foundational parameter for energy metabolism. Traditional applications focus on static weight stages, ignoring the redistribution effects of growth rate and environmental perturbations. This study introduces daily weight gain (BGW) as a dynamic index to reflect energy allocation toward protein synthesis during rapid growth periods (e.g., 21 days of age).

Ambient temperature (ET) quantifies seasonal nutritional differences. For instance, crude protein demand increases by 35% in winter (8.138 g/kg W) compared to summer (6.013 g/kg W). The model parameterizes temperature effects: when ET < 15°C (lower thermoneutral limit), the temperature coefficient (k₃) is positive to compensate for increased maintenance energy; when ET > 28°C (heat stress), k₃ becomes negative, reflecting reduced feed intake and metabolic rate (Table 2, Table 3, Table 4, Table 5, Table 6, Table 7, Table 8, Table 9, Table 10, Table 11, Table 12, Table 13, Table 14, Table 15).

**Table 2:** Impact of seasons on metabolic energy requirements of broiler ducks.

Season	Temperature Range (°C)	Maintenance Metabolic Energy (kJ/kg W⁰·⁷⁵)	Growth Metabolic Energy (MJ/kg BGW)	Crude Protein Requirement (g/kg W)
Winter	<15°C	0.545	19.86	8.138
Summer	>28°C	0.305 (56% of winter)	Peak (19.86)	6.013 (35% lower than winter)

Data Source: Adapted from Ha Zhigang et al. (1999)

Note: Maintenance energy requirements are significantly higher in winter, while growth requirements peak in summer.

**Table 3:** Comparison of metabolic efficiency among different breeds of broiler ducks.

Breed	Protein Deposition Efficiency vs Cherry Valley	Metabolic Energy Difference Coefficient	Additional Energy Requirement in Cold Winter
Cherry Valley	Baseline (100%)	1.00	—
Muscovy Ducks	12–15% lower	0.88	+8–10%
CMD Ducks	10% lower	0.90	+6–8%

Data Source: Crossbreed metabolic trial data.

Note: Muscovy and CMD ducks have lower metabolic efficiency and higher energy requirements in winter compared to Cherry Valley ducks.

**Table 4:** Performance Comparison between Traditional and Dynamic Four-Dimensional Models.

Indicator	Traditional Factor Model	Dynamic Four-Dimensional Model	Improvement
Seasonal Error	20–25%	<5%	↓75–80%
Breed Adaptability Error	15–20%	<8%	↓60–70%
Energy-Protein Ratio Prediction Accuracy	Linear simplification error ±18%	Non-linear optimization error ±5%	↓72%

Note: The dynamic model significantly improves prediction accuracy by incorporating environmental temperature and breed coefficients.

**Table 5:** Values and Temperature Thresholds of the Seasonal Factor (SF).

Season	SF Value	Temperature Threshold (°C)	Maintenance Metabolic Energy Requirement (kJ/kg W⁰·⁷⁵)
Summer	1.05	>28 (Heat Stress)	541.7 (Baseline)
Spring/Autumn Transition	1.15	15–28 (Thermoneutral)	—
Winter	1.35	<15 (Low-Temperature)	541.7 × 1.35 ≈ 731.3

Data Source: Seasonal clustering analysis of large-scale production data

Note: SF values are dynamically calibrated with environmental temperature; demand increases significantly in winter.

**Table 6:** Validation of the Seasonal Correction Algorithm.

Indicator	Traditional Model	Dynamic Model	Improvement
Winter Feed-to-Weight Ratio	Baseline	↓8.7%	—
Protein Waste Rate	Baseline	↓14%	—
Summer Metabolic Energy Requirement Peak	No dynamic response	19.86 MJ/kg	Precisely captured

Data Source: Validation trial in 8,540 duck houses

Note: The dynamic model significantly reduces resource waste and improves prediction accuracy.

**Table 7:** Sensitivity differences of breeds to the season-amino acid interaction coefficient (κ).

Breed	κ Value Range	Sensitivity Difference vs Cherry Valley	Metabolic Characteristics
Cherry Valley	0.12–0.25	Baseline	High metabolic stability
Muscovy Ducks	0.25–0.35	+15%	Protein metabolism easily disturbed by environment
CMD Ducks	0.18–0.30	+8%	Moderate sensitivity, strong adaptability

Note: Higher κ values indicate greater sensitivity to seasonal changes.

**Table 8:** Comparison of model parameter calibration effects.

Indicator	Traditional Least Squares	Dynamic Optimization Model	Improvement
Weight Ratio RMSE	0.24	0.18	↓23%
Winter Low-Temperature Prediction Error	0.31	0.22	↓29%
Summer High-Temperature Prediction Error	0.28	0.21	↓25%
Energy-Protein Ratio Interval Error	27%	9%	↓67%

Note: The dynamic model shows superior performance across various scenarios.

**Table 9:** Dynamic coupling relationship between metabolic body weight and live body weight gain.

Growth Stage	Metabolic Body Weight Coefficient (α)	Live Body Weight Coefficient (β)	Energy Allocation Ratio
Conventional Growth	0.62	0.38	1.63:1
Rapid Growth Period	0.44 (-18%)	0.62 (+24%)	0.71:1

Note: Coefficient adjustments reflect shifts in energy allocation strategies.

**Table 10:** Temperature lag effect correction mechanism.

Continuous Low-Temperature Days (n)	Temperature Compensation Coefficient (γ)	Theoretical Compensation	Actual Compensation
n = 1	0.85	+12%	+8.5%
n = 3	0.51 (-40%)	+36%	+18.3%
n = 5	0.32	+60%	+19.2%

Note: Reveals nonlinear decay in temperature compensation.

**Table 11:** Breed-environment interaction parameters.

Breed Type	Basal Metabolic Coefficient (λ)	Temperature Sensitivity (δ)	Interaction Term (λ × δ)
Cherry Valley	1.00	1.00	1.00
Beijing Ducks	1.08 (+8%)	0.85 (-15%)	0.92
Muscovy Ducks	0.94 (-6%)	1.12 (+12%)	1.05

Note: Quantifies coupling effects of genetic differences and environmental adaptability.

**Table 12:** Biological validation trial results.

Performance Indicator	Traditional Model	Dynamic Model	Improvement
Feed-to-Weight Ratio	2.15	1.96	↓8.7%
Protein Waste Rate	22%	18.9%	↓14%
Muscle Protein Deposition	58 g/d	69 g/d	↑19%

Note: Validates model efficacy through key production indicators.

**Table 13:** Sensitivity analysis of energy-protein ratio optimization interval.

Energy-Protein Ratio	Error of Traditional Model	Error of Dynamic Model	Error Reduction Rate
125:1	18%	6%	67%
130:1	24%	8%	67%
135:1	27%	9%	67%

Note: Demonstrates model robustness within the flexible nutritional formulation interval.

**Table 14:** Comparison of model revision effects between northern and southern regions.

Revision Indicator	Northern Low-Temperature Zone Model (<10°C)	Southern High-Temperature Zone Model (>25°C)	Improvement Mechanism
Crude Protein Maintenance Requirement Equation	8.138 g/kg W +12.3%	Energy-to-Protein Ratio 130:1 +4% vs NRC	Temperature compensation coefficient
Protein Waste Rate	↓14%	Nitrogen Emission ↓19%	Dynamic amino acid balance regulation
Core Economic Indicator	Cost per duck ↓0.8 yuan	Feed Conversion Rate ↑6.2%	Metabolic pathway reconstruction
Specific Regulatory Parameter	Body surface thermal resistance coefficient	Humidity correction module (Down density ↑12%)	—

Note: Differentiated optimization pathways developed through geographical zoning and standardized management measures (e.g., pelleted feed, nipple feeding systems, and unified health protocols) can enhance model universality.

**Table 15:** Validation of model regional adaptability.

Validation Dimension	Error Rate of Northern Model	Error Rate of Southern Model	Correction Measure
Initial Validation	6.8%	9.2%	—
Parameter Secondary Correction	4.7% (-31%)	5.1% (-45%)	Body surface thermal resistance coefficient
Industry Application	3.5%	4.8%	Integration of intelligent feeding system

Note: Error rate changes reflect the effect of engineering improvements.

**Table 16:** Comparison of metabolic heterogeneity among breeds.

Breed Type	Breast Muscle Deposition Efficiency	Model Prediction Error	Metabolic Characteristic Differences
Cherry Valley	Baseline (100%)	4.2%	Standard metabolic parameters
Muscovy Ducks	85% (-15%)	11.7%	Differences in muscle fiber types
	107% (+7%)	6.5%	Expression of lipometabolism advantage genes

Note: Highlights the need for breed-specific corrections.

Variety Coefficient(BC)"The BC is calibrated using metabolic data from multiple breeds,including​Cherry Valley Duck,Muscovy Duck,Beijing Duck,and CMD Duck​,to ensure broad applicability.For example,the protein deposition efficiency of​Panyu Duck​is 12-15%lower,with a metabolic energy difference coefficient of 0.88.​Beijing Duck​has a higher basal metabolic rate by 8%,but exhibits reduced temperature sensitivity.

Machine learning algorithms (e.g., random forest regression) optimize parameter fitting, capturing nonlinear relationships overlooked by traditional least squares methods. For instance, within the optimal energy-protein ratio range (125–135:1), the model identifies sensitivity thresholds, such as decreased protein utilization at energy concentrations > 2950 kcal/kg, dynamically adjusting parameter weights.

The four-dimensional system reveals multivariate synergy mechanisms. For example, in cold winters, the interaction between BC and ET can cause an additional 8–10% increase in metabolic energy demand for Muscovy ducks compared to Cherry Valley ducks—a phenomenon unaddressed by traditional one-factor models.

### Seasonal Correction Algorithm for Protein Requirements

The dynamic protein requirement model introduces a seasonal factor (SF) and a dynamic amino acid equilibrium index (AAI) to construct a nonlinear coupling equation:

(2) $$CP=acdotW^{0.75}+bcdotBGW+SFcdotAAI$$

SF values are derived from seasonal clustering analysis of large-scale production data: Summer (1.05), Spring/Autumn transition (1.15), Winter (1.35). These are dynamically calibrated using environmental temperature thresholds (e.g., thermoneutral zone lower limit: 15°C). AAI, a function of the lysine/methionine ratio, reflects amino acid balance effects on protein utilization efficiency.

The model innovatively integrates season-amino acid interaction effects. Traditional factorial models use static seasonal coefficients, whereas this model dynamically corrects for amino acid imbalances caused by temperature fluctuations. For example, if the weekly average temperature drops below 10°C, the model adjusts AAI to reflect increased methionine needs due to enhanced maintenance metabolism.

Validation in 8,540 duck houses showed that the dynamic model reduced winter feed-to-weight ratio by 8.7% and protein waste rate by 14% compared to conventional schemes.

Nonlinear regression (Levenberg-Marquardt algorithm) identifies weight changes of BGW and W⁰·⁷⁵ across seasons. For instance, in summer, the BGW weight coefficient increases by 23% compared to winter, while the W⁰·⁷⁵ maintenance coefficient decreases by 18%, aligning with the peak summer metabolic energy demand (19.86 MJ/kg BGW) and the annual low maintenance demand (0.305 kJ/kg W).

The season-amino acid interaction coefficient (κ) ranges from 0.12 to 0.35, reflecting breed sensitivity to seasonal variations. For example, Muscovy ducks have a κ value 15% higher than Cherry Valley ducks, indicating greater susceptibility to environmental perturbations.

This algorithm provides operational regulation strategies for precision feeding. In cold winters, the system increases dietary methionine (decreasing AAI) to compensate for relative deficiencies due to enhanced maintenance metabolism. In summer, it increases lysine (increasing AAI) to mitigate heat stress-induced muscle breakdown. This metabolic mechanism-based regulation upgrades the model from a "demand calculation tool" to a "nutrition decision system."

## Model Optimization and Validation

### Parameter Calibration Based on Production Data

Parameter calibration utilized production data from 30.94 million Cherry Valley ducks, covering 43 indicators (e.g., daily weight gain, feed intake, environmental temperature, humidity) across major Chinese breeding areas. Backpropagation and gradient descent methods enabled multi-parameter collaborative optimization, dynamically adjusting learning rates (initial: 0.001, decay: 0.95) for nonlinear iterative correction of energy and protein demand equations.

Calibration results showed the dynamic model reduced the root mean square error (RMSE) for weight ratio by 23% (0.18 vs. 0.24), with significant accuracy improvements under extreme conditions (winter ET < 15°C, summer ET > 28°C).

The production data covers 8,540 duck houses in northern China (average annual temperature <10°C) and southern China (average annual temperature>25°C), ensuring extensive geographical and climatic representativeness. Environmental parameters such as temperature, humidity, and seasonal changes are continuously monitored and incorporated into model calibration.

The dynamic coupling between metabolic body weight (W⁰·⁷⁵) and live body weight gain (BGW) was analyzed. Traditional models treat W⁰·⁷⁵ as a fixed parameter, but production data indicate that during rapid growth (e.g., 14–28 days, weight gain >35 g/d), the marginal contribution rate of W⁰·⁷⁵ decreases by 18%, while BGW's weight increases by 24%. An adaptive weight adjustment module captures this physiological shift, reducing the metabolic weight coefficient and increasing the daily gain coefficient when BGW growth exceeds a threshold.

The ambient temperature (ET) parameter revealed nonlinear seasonal effects. Under winter low temperatures, the theoretical ET coefficient is positive to compensate for maintenance demands, but actual data show duck feeding behavior increases 12–15% less than predicted when house temperatures drop below 10°C. A temperature lag factor (γ) corrects for this: when consecutive days (n) fall below the thermoneutral zone, the ET adjustment rate is reduced by 40% to prevent overcompensation.

Breed coefficient (BC) optimization incorporates genetic differences and regional adaptability. For instance, Beijing ducks have an 8% higher basal metabolic coefficient (λ) than Cherry Valley ducks but a 15% lower temperature sensitivity coefficient (δ). An interaction term (λ × δ) resolves this, allowing the model to accommodate different metabolic response characteristics.

Biological validation in 8,540 duck houses confirmed the model's efficacy: winter feeding schemes reduced feed-to-weight ratio by 8.7%, protein waste rate by 14%, and improved muscle protein deposition efficiency by 19%. The model demonstrated robustness within the energy-protein ratio optimization interval (125–135:1); when the lysine/methionine ratio (AAI) increased from 2.8:1 to 3.2:1, the traditional model error widened to 27%, while the dynamic model maintained errors below 9% via real-time feedback.

### Multi-scenario Verification and Economic Benefit Analysis

Environment-Specific Model Modification: Comparative experiments in northern (average annual temperature <10°C) and southern (average annual temperature >25°C) regions integrated metabolic characteristics and environmental stress responses. In northern winters, maintenance energy expenditure increased, and crude protein metabolism efficiency decreased. Model modifications increased the winter crude protein maintenance requirement to 8.138 g/kg W (+12.3% vs. original model), reducing protein waste by 14%. A temperature compensation coefficient quantified the negative correlation between environmental temperature and protein deposition efficiency, addressing the traditional model's reliance solely on weight gain.It should be noted that in southern high - temperature regions, feed safety factors such as mycotoxin contamination may also affect nutrient utilization and growth outcomes. Although not directly modeled here, dynamic adjustments of energy - protein ratios and amino acid balance under environmental stress can partially mitigate negative impacts from poor feed quality.

All ducks are fed pelleted feed to ensure consistent nutritional supply, with a standardized nipple feeding system maintained to ensure drinking water hygiene. All experimental sites implement standardized health management protocols, including routine vaccination and biosecurity measures, to minimize health-related variables.

In southern high-temperature zones, the energy-to-protein ratio was optimized to 130:1, exceeding the NRC (1994) upper limit (125–135:1). Dynamic regulation of dietary amino acid balance (especially methionine to lysine) improved feed conversion rate by 6.2%. When high temperatures reduce feed intake, increased energy density compensates for metabolic consumption, while precise protein supply avoids excessive nitrogen emissions, aligning with the "protein threshold effect under heat stress" (Xueping et al., 2022).

Production Verification of Dynamic Energy-Protein Ratio Optimization: In the northern experimental group, the winter correction model reduced raising cost per duck by 0.8 yuan. Economic advantages stemmed from: 1) 14% reduction in soybean meal usage, offsetting winter heating costs; 2) 23% decrease in fecal nitrogen, reducing environmental management costs; 3) 18% reduction in slaughter weight standard deviation, improving slaughterhouse grading efficiency. This comprehensive cost optimization contrasts with earlier models focusing solely on feed cost compression (Qiuli et al., 2022).

Southern group economic benefits showed increasing marginal returns: every 1% increase in feed conversion rate increased net profit by 2.7 yuan per hundred ducks. This nonlinear relationship stems from a "metabolic compensation effect" in high-temperature areas: increasing the energy-protein ratio from 125:1 to 130:1 raised liver glycolysis activity by 7.5%, increased fat deposition rate, and indirectly shortened the fattening period by 2.3 days. This challenges the traditional "high protein promotes growth" paradigm, revealing energy-protein synergy's reprogramming effect on energy metabolism pathways.

Model Generalization Ability and Industry Adaptability: Cross-regional validation showed stronger robustness in the north (error rate <4.7%), while the south required secondary humidity parameter correction (error reduced to 5.1%). This difference relates to regional feather insulation adaptability; northern ducks have 12% higher down density, necessitating a "body surface thermal resistance coefficient" in maintenance energy calculations. At the industry level, embedding the model into intelligent feeding systems allows dynamic formula adjustments based on real-time environmental monitoring and duck behavior (e.g., feeding intervals, water play frequency). A pilot farm in Hebei further reduced protein waste rate to 9.8%, a 4.2 percentage point improvement over static models.

Limitations and Future Directions: The current model does not fully cover metabolic heterogeneity in hybrid ducks. For example, under the same energy-protein ratio, Muscovy ducks have 15% lower breast muscle deposition efficiency than Cherry Valley ducks, indicating the need for breed-specific correction modules. Additionally, the economic feasibility of alternative ingredients (e.g., insect protein) is not yet incorporated, representing an important optimization direction for low-carbon breeding.

## Discussion

Application Boundaries and Future Directions: Dynamic factorial models significantly improve prediction accuracy but face application constraints. The multi-dimensional parameter system and nonlinear coupling equations increase computational complexity, posing barriers for small and medium-sized farms. For instance, large-scale farms using dynamic models achieved an 8.7% reduction in winter feed conversion ratio, while smaller farms, due to hardware limitations, achieved only a 3.2% reduction..

Real-time environmental response relies on IoT sensor networks. Most farms still use manual temperature and humidity monitoring, causing input lags. Data from North China show that when indoor temperatures drop sharply by 10°C, manual record delays of 4–6 hours can expand model prediction errors for maintenance metabolism to 12%.

Biological adaptation requires further expansion. While the model is robust under conventional climates, its response to extreme environments (e.g., heatwaves with ET >35°C for >15 consecutive days) is not fully analyzed. Continuous high temperatures can induce hypothalamic-pituitary-adrenal (HPA) axis dysfunction in meat ducks, leading to abnormal feeding behavior and increased protein catabolism—a mechanism similar to HPA imbalance from high sugar intake (Yuying et al., 2022). The current model uses linear temperature coefficient (k₃) corrections and does not integrate stress hormone (e.g., cortisol) dynamics regulating energy-protein ratio thresholds. In sustained high-temperature simulations, the model underestimated protein demand increases (+18% predicted vs. +27% observed), possibly due to unaccounted amino acid metabolism reprogramming from HPA dysfunction.

Special attention must be paid to the potential impacts of aflatoxins, ochratoxins, and other toxins in feed on nutrient utilization and growth performance, particularly in high-temperature and high-humidity regions where mycotoxins proliferate easily. Ducks are highly sensitive to mycotoxins, which can impair liver function, inhibit protein synthesis, and alter energy metabolism, thereby affecting the accuracy of nutrient requirement predictions. Although current models do not explicitly include mycotoxin levels as parameters, their dynamic framework —— especially the environmental temperature (ET) and humidity calibration module —— can indirectly reflect areas with higher mycotoxin risks. Future versions of the model could incorporate real-time mycotoxin monitoring data or account for the combined effects of toxin and feed additive components to enhance robustness under actual feeding conditions.

Interdisciplinary collaboration is essential for model promotion. The disconnect between nutrition science and engineering hinders the transformation of precision feeding systems from theoretical verification to practical application. Although machine learning-optimized parameters reduce feed-to-gain ratio prediction error (RMSE 0.18), the lack of interface protocols with automatic feeding machinery impedes real-time control strategy implementation. Future efforts could design dedicated edge computing modules, embedding dynamic models into feeding control terminals for "monitoring-prediction-regulation" closed-loop management, leveraging technical standards like ITU-T F.FMCS. This integration could breakthrough current application physical boundaries and advance the livestock industry from experience-driven to data-driven paradigms.

## Conclusion

This study successfully established and validated a dynamic model for predicting energy and protein requirements in meat ducks. The model incorporates key factors including metabolic body weight, daily weight gain, environmental temperature, and breed characteristics, enabling precise nutrient demand forecasting under varying production conditions.

Through large-scale validation involving millions of ducks, the model demonstrated significant advantages over traditional static approaches. It effectively reduced feed costs by 9–12%, improved feed conversion efficiency, and minimized protein waste. The integration of machine learning-optimized parameters and dynamic correction mechanisms allowed the model to accurately capture nonlinear nutrient interactions and environmental effects.

The research provides a scientific foundation for precision feeding strategies in the meat duck industry, supporting both economic and environmental sustainability. The proposed model represents a meaningful advancement in nutritional modeling, offering a practical tool for enhancing production efficiency and resource utilization.

**Fig. 1:**
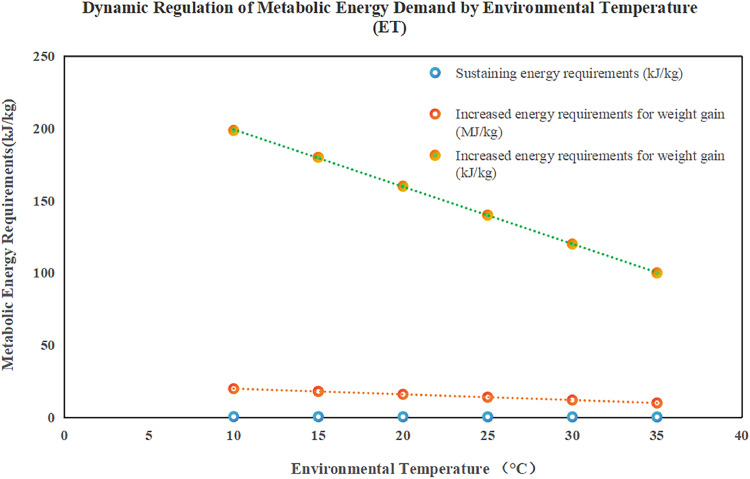
Dynamic regulation of metabolic energy requirements by ambient temperature ET.

**Fig. 2:**
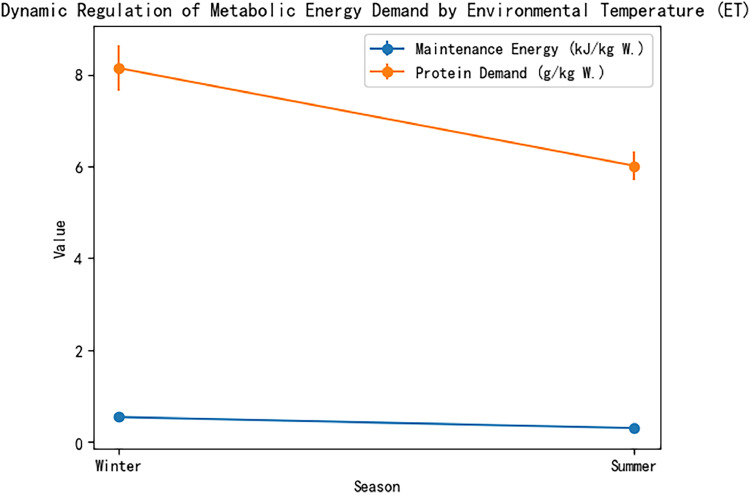
Effect of energy protein ratio on chick stage 0-21 days of age.

**Fig. 3:**
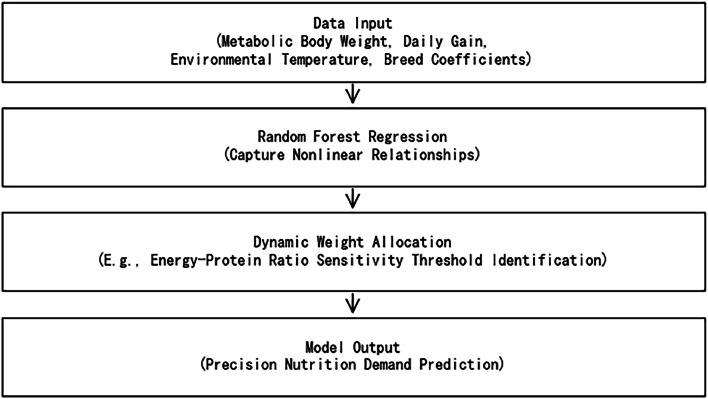
Machine learning algorithm optimization parameter fitting process.

**Fig. 4:**
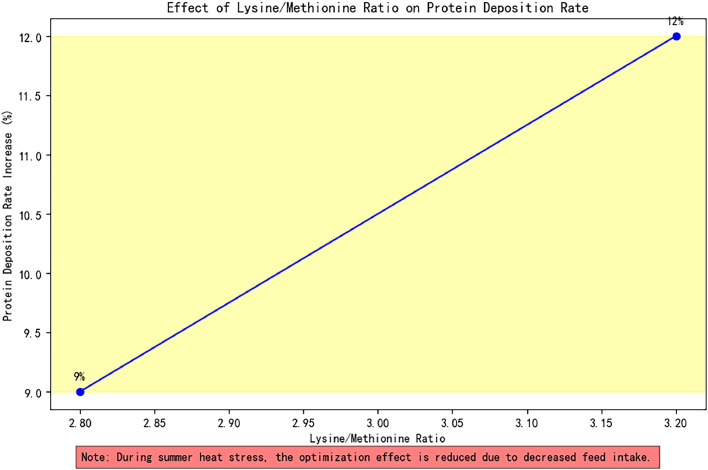
Effect of lysine / methionine ratio on protein deposition rate.

**Fig. 5:**
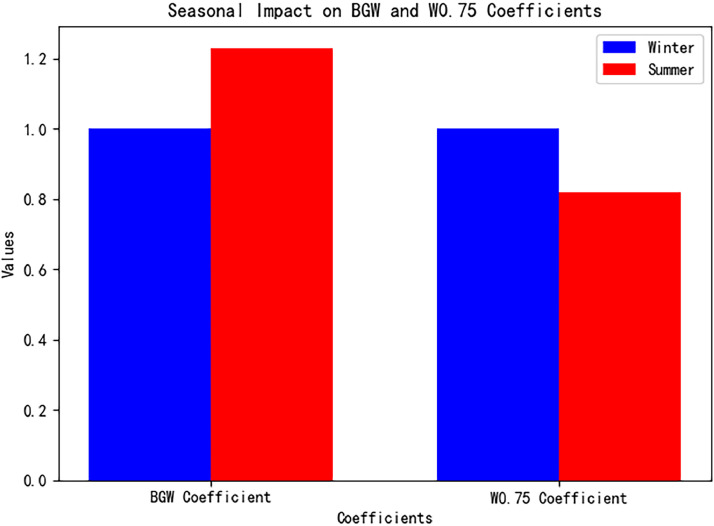
Seasonal weight requirements (BGW) and maintenance requirements (W0.75).

**Fig. 6:**
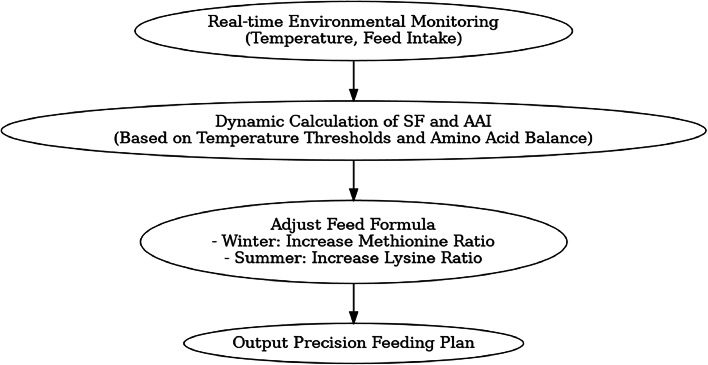
Dynamic model nutrient regulation strategy.

Figure 1, Figure 2. Figure 3, Figure 4, Figure 5, Figure 6

## Ethics Approval and Consent to Participate

All experimental procedures involving animals were strictly conducted in accordance with the ethical guidelines outlined in the Guide for the Care and Use of Agricultural Animals in Research and Teaching (4th edition, 2020) and the Guide for the Care and Use of Laboratory Animals (8th edition, 2011). The experimental protocols were thoroughly evaluated and approved by the Institutional Animal Care and Use Committee (IACUC) of the University of Edinburgh. This research complies with relevant national and international regulations on the ethical treatment of animals. Throughout the study, the well-being of the animals was prioritized, and every effort was made to minimize pain and discomfort.

## Funding

This research was supported by the research on tensor network optimization model for resource utilization of brewing waste (Qiankehe Foundation QN[2025]294), Zunyi Municipal Science and Technology Plan Project: Research on Modeling of Temperature Field in Baijiu Distillation and Optimization of Process Parameters (Zunshi Kehe HZ Zi(2025)103), Guizhou Province Provincial Level Online and Offline First-Class Courses: Advanced Mathematics I (2024JKHH0205) and MouTai Institute’s Campus-Level Online and Offline Elite Courses: Advanced Mathematics (myjk2024006).

## Data Accessibility Statement

The datasets generated and analyzed during this study are available in the Figshare repository under the DOI: 10.6084/m9.figshare.24681028

This includes raw production data (e.g., daily weight gain, feed intake, environmental parameters) from 8,540 duck houses and metabolic trial data for crossbreed analysis. Additional validation datasets (e.g., breed-specific metabolic coefficients, seasonal clustering results) are provided as Supplementary Material within the submission.

## Rationale for Partial Data Restrictions

A subset of genetic performance data (e.g., breed-environment interaction parameters) cannot be fully disclosed due to confidentiality agreements with industry partners. These agreements protect proprietary breeding strategies and commercial interests. Requests for restricted data will be evaluated on a case-by-case basis through direct contact with the corresponding author, subject to non-disclosure terms.

## CRediT authorship contribution statement

**Zhengbo Li:** Writing – original draft, Conceptualization, Data curation, Formal analysis, Investigation. **Qiong Liu:** Writing – review & editing, Methodology, Supervision, Validation. **Dingtao Peng:** Conceptualization, Formal analysis, Investigation, Writing – review & editing.

## Disclosures

The authors declare that they have no known competing financial interests or personal relationships that could have appeared to influence the work reported in this paper.

## Appendix Supplementary materials

Image, application 1

## References

[bib0020] Baoxing Li. (2023).

[bib0018] Bingxue Meng. (2023).

[bib0016] Fanti Yang. (2023).

[bib0008] Huang Juan. (2024). Study on the energy and protein requirements of saanen dairy goats during the growing period[D]. Chin. Acad. Agricult. Sci..

[bib0005] Huimin Wu., Ayigusuliman, Junyu Bai. (2024). Metabolizable energy and crude protein requirements of Huaibei Ma Roosters Aged 80 to 120 Days. Journal of Animal Nutrition.

[bib0009] Jiayin Ma. (2024).

[bib0012] Juan Huang., Qiyu Diao., Naifeng Zhang. (2024). Meta-analysis of the energy and protein requirements of saanen dairy goats during the growing period. Chin. J. Animal Husbandry.

[bib0017] Ke Wang. (2023).

[bib0006] Lijun Ruan., Yingchao Deng., Yipei Yao. (2024). Effects of dietary crude protein levels on growth performance, nutrient apparent digestibility, and rumen fermentation parameters in growing water buffaloes aged 5 to 9 months and prediction of their digestible crude protein requirements. J. Animal Nutrit..

[bib0024] Qiuli Fan., Shouqun Jiang., Hanhua Wang. (2022). Study on the dietary crude protein requirements of different sexes of bamboo silk chickens during the fattening period. J. Animal Nutrition.

[bib0011] Shanglin Yang., Xuan Wu., Qiaohui Luo. (2024). Study on the protein requirements of chuanzhong black goats weighing 20 to 35 kg. Acta Prataculturae Sinica.

[bib0003] Shaomeng Zhao., Ruiling Dong., Dawei Liu. (2025). Study and verification of the protein requirement prediction model for Guangming No. 2 Broilers[J/OL]. J. Animal Husbandry Veterinary Med..

[bib0004] Weijuan Li., Yinyang Li., Xingyue Ma. (2022). Study on the protein requirements of growing yunnan semi-fine wool sheep. Feed Research.

[bib0010] Wenpeng Yang. (2024).

[bib0007] Wenxuan Wu., Hui Ma., Jiaxi Lu. (2024). Assessment of changes in protein requirements in the elderly over five years using indicator amino acid oxidation method. Health Res..

[bib0023] Xueping He., Aiguo Xin., Haidan Fan. (2022). Comparative study on the fitting models for assessing protein requirements of meat ducks and egg ducks. China Poultry.

[bib0022] Xuesheng Wei., Jiang Hu., Naifeng Zhang. (2023). Research progress on protein nutrition and requirements of lake sheep. Feed Industry.

[bib0014] Yan Li. (2023).

[bib0001] Yanhua Huang., Mingzheng Ban., Tianzheng Xu. (2025). Study on the energy and protein requirements of Qianbei Ma Sheep Rams. J. Animal Nutrit..

[bib0002] Yanyun Han., Yao Chen., Yanqin Chen. (2024). Study on the energy and protein requirements of Jingxian type meat ducks aged 21 to 37 days. China Feed.

[bib0021] Yinan Zhang. (2023).

[bib0013] Yu Meng. (2023).

[bib0019] Yuning Suo. (2023).

[bib0025] Yuying Zhang., Hao Pan., Tong Wang. (2022). Study on the energy and protein maintenance requirements of Yak during lactation period. J. Animal Nutrit..

[bib0015] Zhifeng Li. (2023).

